# Treatment patterns and survival of low and intermediate‐risk prostate cancer in end‐stage kidney disease: A retrospective population cohort study

**DOI:** 10.1002/cam4.5571

**Published:** 2023-01-16

**Authors:** Nagaraju Sarabu, Weichuan Dong, Al W. Ray, Austin Fernstrum, Megan Prunty, Lee E. Ponsky, Jonathan E. Shoag, Vahakn B. Shahinian, Krista L. Lentine, Siran M. Koroukian

**Affiliations:** ^1^ Division of Nephrology University Hospitals Cleveland Medical Center Ohio Cleveland United States; ^2^ Population and Quantitative Health Sciences Population Cancer Analytics Shared Resource, and the Case Comprehensive Cancer Center, Case Western Reserve University Ohio Cleveland United States; ^3^ Department of Urology University Hospitals Cleveland Medical Center Ohio Cleveland United States; ^4^ Division of Nephrology, Department of Medicine University of Michigan Michigan Ann Arbor United States; ^5^ Center for Abdominal Transplantation Saint Louis University School of Medicine Missouri St. Louis United States

**Keywords:** disparity, end‐stage kidney disease, kidney transplant, prostate cancer, shared decision making, treatment

## Abstract

**Background:**

In accordance with guidelines, observation with or without active surveillance for low‐risk prostate cancer increased in recent years in the general population. We compared treatment patterns and mortality for low‐ and intermediate‐risk prostate cancer and mortality rates among end‐stage kidney disease (ESKD) and non‐ESKD patients.

**Methods:**

This is a retrospective population‐based observational cohort study of Surveillance, Epidemiology, and End Results‐Medicare data of men aged 66 years and older with localized prostate cancer (2004–2015). ESKD status was determined using Medicare billing codes. Multivariable logistic regression models and Cox‐proportional hazards models were used to study definitive treatment patterns and mortality, respectively.

**Results:**

For low‐risk prostate cancer, dialysis patients (N = 83) had lower but not statistically significant odds (OR, 0.74; 95% CI: 0.48–1.16) of receiving definitive treatment than non‐ESKD patients (*N* = 24,935). For those with intermediate‐risk prostate cancer, dialysis patients (N = 254) had lower odds to receive definitive treatment (OR, 0.54; 95% CI: 0.42–0.72) than non‐ESKD patients (*N* = 60,883). From 2004–2010 to 2011–2015, for patients with low‐risk prostate cancer, while the receipt of definitive treatment for non‐ESKD patients trended down from 72% to 48%, it trended up for dialysis patients from 55% to 65%. Kidney transplant patients (*N* = 33 for low‐risk and *N* = 91 for intermediate‐risk) had lower rates of definitive treatment for low‐risk and similar rates of treatment for intermediate‐risk prostate cancer compared to non‐ESKD patients.

**Conclusions:**

The disparity in definitive treatment rates for low‐risk prostate cancer among dialysis patients exists despite their high mortality, compared to the general population.

## Graphical abstract

In contrast to the general population, though older dialysis patients have a three times higher mortality, they receive definitive treatments for low‐risk prostate cancer at high rates and these rates have increased in the recent era. Kidney transplant patients do not experience higher prostate cancer deaths in the low‐risk prostate cancer than the general population and hence immunosuppression does not need to sway providers toward definitive treatments.

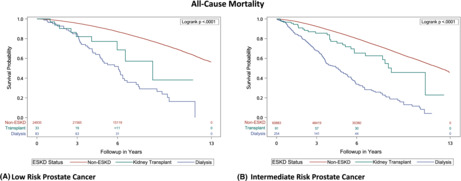

## 1 INTRODUCTION

Prostate cancer (PCa) is a common malignancy in men with end‐stage kidney disease (ESKD). National Comprehensive Cancer network (NCCN) provides a risk framework, ranging from very low to very high‐risk PCa, to help clinicians in the shared decision‐making process for management.^1^ Life expectancy is a major factor that goes into this shared decision‐making and accordingly, vulnerable populations such as those with ESKD need to be managed after careful consideration of their life expectancy. ESKD is defined as dialysis dependency or receipt of a kidney transplant (KT), both of which are associated with reduced life expectancy compared to the general population. For example, a 55‐year‐old man living in the United States (US), who is on chronic dialysis has a life expectancy of 6.9 years, compared to 23.8 years for a 55‐year‐old man from the general population and his counterpart with KT has a much better life expectancy of 17.3 years, though lower than that of the general US population.^2^

In the general population, for low‐risk PCa, active surveillance (AS), where laboratory, radiographic, and prostate biopsies are performed at regular intervals to monitor progression and watchful waiting (WW), which is less intensive than AS and involves symptomatic monitoring, are strongly recommended as opposed to immediate definitive treatment (prostatectomy or radiation) for patients with >10 years and < 10 years of life expectancy, respectively.^3^, ^4^, ^5^ Given that the life expectancy of ESKD patients is much lower than that of the general population, one would expect that their low‐risk PCa would be managed with AS or WW for low‐risk PCa at higher rates than non‐ESKD patients. However, a subset of dialysis patients with PCa, who are getting evaluated for KT poses an additional layer of complexity relating to a theoretical concern of immunosuppression after KT worsening prognosis of PCa,^6^, ^7^, ^8^ and hence may prompt immediate definitive treatment and potentially delay a rather life‐saving KT.^9^

In this study, we sought to compare rates of definitive treatment (prostatectomy or radiation) for low‐risk and intermediate‐risk PCa between ESKD and non‐ESKD patients using the Surveillance, Epidemiology, and End Results (SEER)‐Medicare database. Our secondary aim was to compare all‐cause mortality between the groups.

### 1.1 Methods

This was a retrospective study of SEER data with linked administrative Medicare claims. The Case Western Reserve University Institute Review Board approved the study with a waiver of informed consent (Protocol #2019–0264). This report follows the Strengthening the Reporting of Observational Studies in Epidemiology reporting guideline for cohort studies.^10^

### 1.2 Data source

The SEER portion of the database includes patient demographics, date of diagnosis, tumor characteristics, and vital status, while the Medicare portion includes enrollment and claims data.^11^ Approximately 27.8% of the US cancer population is captured in SEER from 18 geographic areas and includes a diverse demographic population that has been previously shown to be similar in characteristics to the US population.^12^, ^13^

### 1.3 Study population

PCa data for years of diagnosis between January 1, 2000, and December 31, 2016, was available for analysis. We included patients with fee‐for‐service participation and enrollment in both Part A and Part B of Medicare, at least 1 year before and 1 year after PCa diagnosis to ascertain dialysis and KT status prior to PCa diagnosis and to capture receipt of treatment after PCa diagnosis. Dialysis and KT patients are eligible for Medicare due to their ESKD status, but the general population is eligible for Medicare when they reach 65 years of age or if they are younger than 65 years of age with a disabling condition. Accordingly, to avoid confounding due to age, our study participants were those who were documented as men, age 66 years and older, who were diagnosed with localized PCa between January 1, 2004 (when SEER started including prostate‐specific antigen (PSA) and Gleason score in the registry) and December 31, 2015.

### 1.4 Exposure and comorbidities

In the year prior to the diagnosis of PCa, billing codes were used to categorize patients as dialysis dependent as described earlier,^14^ or having a KT (Appendix S1). If a given patient had procedure codes for both dialysis or KT, they were categorized as dialysis or KT based on whichever codes occurred the latest, prior to the diagnosis of PCa. If patients had no codes for dialysis or KT, they were categorized as non‐ESKD. Patients who were dialysis‐dependent at the onset of the study but received a KT during the follow‐up period were included in the dialysis cohort. We used the chronic condition segment of the Medicare Beneficiary Summary File to assess comorbidities.

### 1.5 Risk group categorization

We used the American Joint Committee on Cancer (AJCC) 6th edition Tumor (T) stage, Gleason score, and prostate specific antigen (PSA) level at diagnosis to categorize patients into low‐ and intermediate‐risk groups based on NCCN definitions.^1^ For this risk categorization, we excluded patients with missing, unknown, or T‐stages other than low‐risk or intermediate risk (see below) (*N* = 44,591), missing PSA (N = 9527), or missing Gleason score (*N* = 3552) data. In the included cases, we defined low‐risk and intermediate‐risk PCa as follows. (1) Low risk: T stages T1c and T2a, Gleason score of 6 or less, or serum PSA level of ≤10 ng/mL, (2) Intermediate risk: T stages T2b and T2c, Gleason score of 7, and serum PSA level of 10–20 ng/mL.

### 1.6 Outcomes

The primary outcome was the receipt of definitive treatment, defined as the receipt of prostatectomy, any form of radiation therapy, or both. The secondary outcome was all‐cause mortality.

### 1.7 Statistical analysis

Baseline characteristics among those with dialysis, KT, and without ESKD were compared by using either analysis of variance or chi‐square test. We evaluated the likelihood of receiving definitive treatment by using logistic regression models and reported results as odds ratios (OR). Covariates included in the multivariable logistic regression model were age at PCa diagnosis, race, socioeconomic status (a proxy for social support: having a significant other versus none; census tract poverty level of where they were residing at the time of diagnosis (>20% of the population vs. ≤20%), living in a metropolitan county versus in a non‐metropolitan county), and comorbidities (e.g., diabetes, hypertension, cardiovascular disease). Since tumor characteristics, PSA, and Gleason score were used to define low and intermediate risk, we did not include these variables in the multivariable models to avoid overfitting. We have also explored how treatment rates differed for patients diagnosed with PCa between the years 2004–2010 and 2011–2015 for each group using the chi‐square test (noting that since 2010, AS or WW has been recommended as first‐line treatment for low‐risk PCa). Time to death from any cause was analyzed separately for low and intermediate risk categories using Kaplan Meier curves to illustrate survival rates up to 13 years and Cox proportional hazards models to quantify the hazard of death (reported as hazard ratio (HR)). Time zero was the date of diagnosis of PCa and all patients were right censored at the study end date (December 31, 2016). Dialysis patients who received KT during follow‐up were also right censored at the same study end date. In the multivariable Cox proportional hazards models, age, race, socio‐economic status, and comorbidities were used for adjustment. Receipt of treatment was also used in the Cox proportional hazards model. Since the number of deaths attributed to PCa was low, we did not conduct any survival analyses for time to PCa‐specific death. Two‐tailed *p*‐values less than 0.05 were considered statistically significant. All analyses were performed using SAS version 9.4 (SAS Institute).

## 2 RESULTS

### 2.1 Baseline characteristics

**FIGURE 1:**
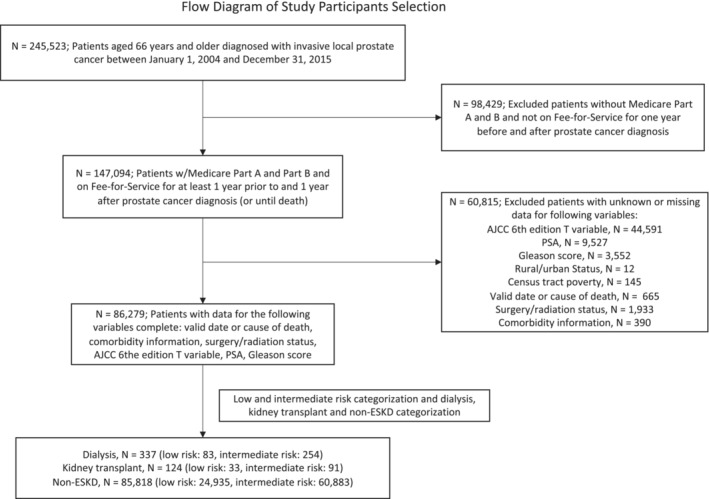
Flow diagram of study participant selection. Low risk: T stages T1c and T2a, Gleason score of 6 or less, and serum PSA level of ≤10 ng/mL and intermediate risk: T stages T2b and T2c, Gleason score of 7, and serum PSA level of 10–20 ng/mL. Billing codes were used to categorize patients as dialysis or having a kidney transplant, as described in supplement material. Others were categorized as non‐ESKD. AJCC, American Joint Committee on Cancer; PSA, prostate specific antigen; ESKD, end stage kidney disease.

Our study population included men, age 66 years and older, diagnosed with low‐ and intermediate‐risk PCa, of which there were 83 dialysis, 33 KT, and 24,935 non‐ESKD patients with low‐risk PCa and 254 dialysis, 91 KT, and 60,883 non‐ESKD patients with intermediate‐risk PCa (Figure 1). In the low‐risk group, compared to non‐ESKD patients, dialysis and kidney transplant patients were somewhat younger (mean age in dialysis and KT was 71 years and 70.5, respectively, vs. 72.1 years in non‐ESKD), and had a higher representation of racial and ethnic minority non‐Whites (48.2% and 39.4% in dialysis and KT, respectively, vs. 16.8% in non‐ESKD); more likely to reside in census tracts with greater than 20% of poverty prevalence (38.6% and 33.3% in dialysis and KT, respectively, vs. 17% in non‐ESKD) or metropolitan counties (91.6% and 93.9% in dialysis and KT, respectively, vs. 85.5% in non‐ESKD), and had a higher prevalence of cardiovascular disease (61.5% and 63.6% in dialysis and KT, respectively, vs. 36.8% in non‐ESKD), hypertension (83.1% and 84.9% in dialysis and KT, respectively, vs. 56.8% in non‐ESKD), and diabetes (50.6% and 60.6% in dialysis and KT, respectively, vs. 22.3% in non‐ESKD). Very similar patterns in baseline characteristics were observed in the intermediate‐risk group (Table 1).

**TABLE 1:** Demographic and Clinical Characteristics at Baseline for Low and Intermediate Riska Prostate Cancer Groups for Men (age ≥ 66) in the SEER‐Medicare Database Diagnosed between 2004 and 2015, stratified by dialysis, kidney transplant, and non‐end stage kidney disease.

Patient characteristic^a^	Low risk^b^				Intermediate risk^b^			
	Dialysis, *N* = 83	Kidney transplant, *N* = 33	Non‐ESKD, *N* = 24,935	*p* ^c^	Dialysis, *N* = 254	Kidney transplant, *N* = 91	Non‐ESKD, *N* = 60,883	*p* ^c^
Age (mean(SD))	71.0 (3.9)	70.5 (3.0)	72.1 (4.5)	0.01	71.8 (5.0)	71.8 (4.2)	73.3 (5.5)	<0.01
Non‐Hispanic White Race	43 (51.8)	20 (60.6)	20,747 (83.2)	<0.01	126 (49.6)	74 (81.3)	48,914 (80.3)	<0.01
Married (vs Single or unknown)	47 (56.6)	28 (84.8)	17,702 (71)	<0.01	154 (60.6)	70 (76.9)	42,700 (70.1)	<0.01
Living in a census tract with > 20% population under the federal poverty	32 (38.6)	11 (33.3)	4243 (17.0)	<0.01	83 (32.7)	<11 (<12.1)	11,230 (18.4)	<0.01
Living in a Metropolitan county	76 (91.6)	31 (93.9)	21,311 (85.5)	0.1118	217 (85.4)	85 (93.4)	51,542 (84.7)	<0.01
Cardiovascular disease^d^	51 (61.5)	21 (63.6)	9163 (36.8)	<0.01	171 (67.3)	53 (58.2)	23,576 (38.7)	<0.01
Hypertension	69 (83.1)	28 (84.9)	14,161 (56.8)	<0.01	216 (85)	69 (75.8)	35,102 (57.7)	<0.01
Diabetes	42 (50.6)	20 (60.6)	5561 (22.3)	<0.01	152 (59.8)	47 (51.6)	14,504 (23.8)	<0.01
Received definitive treatment	48 (57.8)	16 (48.5)	16,174 (64.9)	0.059	176 (69.3)	73 (80.2)	49,029 (80.5)	<0.01

Abbreviations: ESKD, end‐stage kidney disease; SEER, surveillance, epidemiology, and end results; SD, standard deviation.

^a^ Age is expressed as mean and standard deviation. Other variables are percentages.

^b^ Low risk: T stages T1c and T2a, Gleason score of 6 or less, and serum PSA level of ≤10 ng/mL and intermediate risk: T stages T2b and T2c, Gleason score of 7, and serum PSA level of 10–20 ng/mL.

^c^ Analysis of Variance for Age differences and chi‐square test for all the other variables.

^d^ Cardiovascular disease includes history of acute myocardial infarction, stroke, congestive heart failure, ischemic heart disease, or atrial fib.

### 2.2 Treatment patterns

Among the 83 dialysis patients with low‐risk PCa, 58% had definitive treatment and out of 33 KT patients with low‐risk PCa, 49% had definitive treatment. By comparison, 65% of non‐ESKD patients with low‐risk PCa received definitive treatment. Both dialysis (OR, 0.74; 95% CI: 0.48–1.16) and KT groups (OR, 0.51; 95% CI: 0.26–1.01) with low‐risk PCa had lower odds of undergoing definitive treatment compared to non‐ESKD patients in univariate models, albeit at not statistically significant levels. This relationship in treatment patterns between dialysis vs non‐ESKD remained the same but for KT vs. non‐ESKD became statistically significant in the multivariable model (Table 2). For dialysis patients who were diagnosed with low‐risk PCa between 2004 and 2010, definitive treatment rates were 54% and trended up to 65% for those diagnosed between 2011 and 2015 (*p*‐value = 0.35). In contrast, for non‐ESKD patients with low‐risk PCa, the treatment rates trended down (72% in 2004–2010 and 48% in 2011–2015, *p*‐value <0.01).

**TABLE 2:** Likelihood of undergoing definitive treatment for low and intermediate risk prostate cancer groups for men (age ≥ 66) in the SEER‐medicare database diagnosed between 2004 and 2015.

Model	Odds Ratio (95% CI) for ESKD Status		
	Non‐ESKD	Dialysis	Kidney transplant
Low risk^a^			
No. of definitive treatments^b^ (%)	16,174 of 24,935 (64.9)	48 of 83 (57.8)	16 of 33 (48.5)
Univariate	1.00 (Referent)	0.74 (0.48 to 1.16)	0.51 (0.26 to 1.01)
ESKD status + Age	1.00 (Referent)	0.70 (0.45 to 1.09)	0.46 (0.23 to 0.92)
ESKD status + Age + Race^c^	1.00 (Referent)	0.70 (0.45 to 1.10)	0.47 (0.23 to 0.93)
ESKD status + Age + Race + Socioeconomic status^d^	1.00 (Referent)	0.75 (0.48 to 1.18)	0.42 (0.21 to 0.84)
ESKD status + Age + Race + Socioeconomic status + Comorbidity^e^	1.00 (Referent)	0.70 (0.45 to 1.10)	0.38 (0.19 to 0.77)
Intermediate risk^a^			
No. of definitive treatments (%)	49,029 of 60,883 (80.5)	176 of 254 (69.3)	73 of 91 (80.2)
Univariate	1.00 (Referent)	0.54 (0.42–0.72)	0.98 (0.60–1.69)
ESKD status + Age	1.00 (Referent)	0.41 (0.31–0.55)	0.76 (0.46–1.34)
ESKD status + Age + Race^c^	1.00 (Referent)	0.47 (0.36–0.63)	0.76 (0.46–1.33)
ESKD status + Age + Race + Socioeconomic status^d^	1.00 (Referent)	0.49 (0.37–0.66)	0.68 (0.40–1.20)
ESKD status + Age + Race + Socioeconomic status + Comorbidity^e^	1.00 (Referent)	0.49 (0.37–0.65)	0.67 (0.40–1.19)

Abbreviations: ESKD, end‐stage kidney disease; SEER, surveillance, epidemiology, and end results.

^a^ Low risk: T stages T1c and T2a, Gleason score of 6 or less, and serum PSA level of <= 10 ng/mL and intermediate risk: T stages T2b and T2c, Gleason score of 7, and serum PSA level of 10–20 ng/mL.

^b^ Definitive treatment = prostatectomy or radiation therapy.

^c^ Race = White and Non‐White.

^d^ Socioeconomic status = having significant other vs. none, living in a census tract with more than 20% people below the federal poverty level versus other, living in a metropolitan county versus in a non‐metropolitan county.

^e^ Comorbidity = Diabetes, hypertension, cardiovascular disease (which is a composite of acute myocardial infarction, stroke, congestive heart failure, ischemic heart disease, and atrial fibrillation).

Among the intermediate‐risk PCa group, 69.3% of dialysis patients had definitive treatment, versus 80.2% in KT patients. By comparison, 80.5% of non‐ESKD patients in the intermediate‐risk PCa had definitive treatment. Compared to the non‐ESKD group, dialysis (OR, 0.54; 95% CI: 0.42–0.72) and KT groups (OR, 0.98; 95% CI: 0.60–1.69) had lower odds of receiving definitive treatment (Table 2). For dialysis patients who were diagnosed with intermediate‐risk PCa between 2004 and 2010, definitive treatment rates were 63% and trended up to 77% for those diagnosed between 2011 and 2015 (*p*‐value = 0.01). In contrast, for non‐ESKD patients with intermediate‐risk PCa, the treatment rates remained unchanged (80% in 2004–2010 and 81% in 2011–2015, *p*‐value = 0.3).

### 2.3 Mortality

**FIGURE 2:**
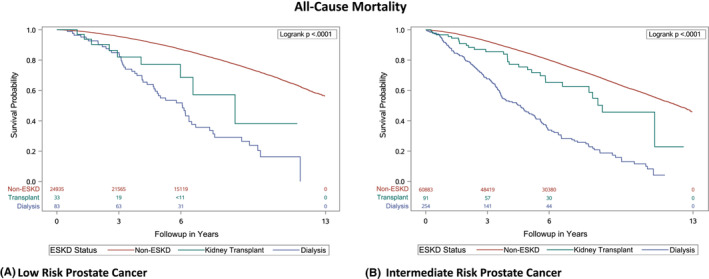
Kaplan Meier Curves for All‐Cause Mortality for Low and Intermediate Risk Prostate Cancer Groups for Men (age ≥ 66) in the SEER‐Medicare Database Diagnosed between 2004 and 2015, stratified by dialysis, kidney transplant, and non‐end stage kidney disease. ESKD, end‐stage kidney disease.

Median follow‐up times were 4.7, 4.1, and 7.1 years for dialysis, KT, and non‐ESKD patients in low‐risk and 3.5, 4.3, 6.0 years for dialysis, KT, and non‐ESKD patients in intermediate‐risk groups. In low‐risk PCa patients, 62.7% and < 33.3% (<11 deaths) died in dialysis and KT populations, respectively, and 21.7% of patients died in the non‐ESKD group. Compared to non‐ESKD patients, dialysis patients had five times the higher hazard of death from any cause (HR, 5.18; 95% CI: 3.94–6.81) and KT patients had three times the higher hazard of death from any cause (HR, 2.77; 95% CI: 1.44–5.32). In the intermediate‐risk PCa group, 63% of dialysis, 32% of KT, and 27% of non‐ESKD patients died. Similar to the association observed in the low‐risk group, dialysis patients had significantly higher mortality than non‐ESKD patients both before and after adjusting for potential confounders (Figure 2 and Table 3).

**TABLE 3:** Hazard of all‐cause mortality for low and intermediate riska prostate cancer groups for men (age ≥ 66) in the SEER‐medicare database diagnosed between 2004 and 2015.

Model	Hazard ratio (95% CI) for ESKD Status		
	Non‐ESKD	Dialysis	Kidney transplant
Low risk^a^			
No. of deaths (%)	5414 of 24,935 (21.7)	52 of 83 (62.7)	<11 of 33 (<33.3)^b^
Univariate	1.00 (Referent)	5.18 (3.94–6.81)	2.77 (1.44–5.32)
ESKD status + Age	1.00 (Referent)	5.94 (4.52–7.80)	3.49 (1.81–6.71)
ESKD status + Age + Race^c^	1.00 (Referent)	5.80 (4.41–7.63)	3.46 (1.80–6.66)
ESKD status + Age + Race^c^ + Socioeconomic status^d^	1.00 (Referent)	5.44 (4.14–7.16)	3.61 (1.88–6.95)
ESKD status + Age + Race^c^ + Socioeconomic status^d^ + Comorbidity^e^	1.00 (Referent)	4.72 (3.59–6.21)	2.84 (1.48–5.47)
ESKD status + Age + Race^c^ + Socioeconomic status^d^ + Comorbidity^e^ + Definitive Treatment^f^	1.00 (Referent)	4.51 (3.43–5.94)	2.69 (1.40–5.18)
Intermediate risk^a^			
No. of deaths (%)	16,439 of 60,883 (27.0)	160 of 254 (63.0)	29 of 91 (31.9)
Univariate	1.00 (Referent)	4.69 (4.01–5.48)	1.78 (1.24–2.56)
ESKD status + Age	1.00 (Referent)	5.38 (4.60–6.28)	2.31 (1.61–3.33)
ESKD status + Age + Race^c^	1.00 (Referent)	5.24 (4.48–6.13)	2.33 (1.62–3.35)
ESKD status + Age + Race^c^ + Socioeconomic status^d^	1.00 (Referent)	5.06 (4.33–5.92)	2.53 (1.75–3.64)
ESKD status + Age + Race^c^ + Socioeconomic status^d^ + Comorbidity^e^	1.00 (Referent)	4.16 (3.56–4.87)	2.22 (1.54–3.19)
ESKD status + Age + Race^c^ + Socioeconomic status^d^ + Comorbidity^e^ + Definitive Treatment^f^	1.00 (Referent)	3.72 (3.18–4.35)	2.15 (1.50–3.10)

Abbreviations: ESKD, end‐stage kidney disease; SEER, surveillance, epidemiology, and end results.

^a^ Low risk: T stages T1c and T2a, Gleason score of 6 or less, and serum PSA level of <= 10 ng/mL and intermediate risk: T stages T2b and T2c, Gleason score of 7, and serum PSA level of 10–20 ng/mL.

^b^ Number is suppressed (*n* < 11) according to the Surveillance, Epidemiology, and End Results cell suppression guidelines.

^c^ Race = White and Non‐White.

^d^ Socioeconomic status = having significant other vs. none, living in a census tract with more than 20% people below the federal poverty level vs. other, living in a metropolitan county vs. in a non‐metropolitan county.

^e^ Comorbidity = Diabetes, hypertension, cardiovascular disease (which is a composite of acute myocardial infarction, stroke, congestive heart failure, ischemic heart disease, and atrial fibrillation).

^f^ Definitive treatment = prostatectomy or radiation therapy.

Due to SEER reporting policies, for outcomes with numbers <11, we report them as “<11”. A small number of KT patients (<11) and 11 dialysis patients died from PCa in low‐ and intermediate‐risk groups combined. In the low‐risk group, 1.2% of non‐ESKD patients died from PCa with a median follow‐up of 6.4 years, while in the intermediate‐risk group, 3.9% of non‐ESKD patients died from PCa with a median follow‐up of 4.5 years.

### 2.4 Kidney transplantation rates after PCa diagnosis among dialysis patients

A small number (<11) of dialysis patients with low‐risk PCa received a KT after a diagnosis of PCa during the follow‐up period, at a median waiting time to KT of 2.3 years (range: 4 months–2.5 years) from the time of PCa diagnosis, and > 75% of these patients received a definitive treatment prior to KT. Among the intermediate‐risk PCa patients on dialysis, a small number (<11) received KT at median waiting times of 1.5 years (range: 1.8 months–3.1 years), and 100% of them received definitive treatment, prior to KT, respectively.

## 3 DISCUSSION

We conducted a retrospective population cohort analysis of the SEER‐Medicare database of patients with low and intermediate‐risk PCa to explore definitive treatment patterns and survival among ESKD patients and observed the following major findings: (1) dialysis patients had five times higher mortality than non‐ESKD patients and despite this, had overall high rates (58%) of prostatectomy or radiation for low‐risk PCa, which was lower than the non‐ESKD group (65%) but was still high given the high mortality rates of dialysis patients. (2) While definitive treatment rates had fallen in the 2011–2015 era compared to the 2004–2010 era in non‐ESKD patients, they have trended up in dialysis patients for low‐risk PCa. (3) Definitive treatments for intermediate‐risk PCa were lower in dialysis patients than in non‐ESKD patients.

Previous studies have shown that localized PCa is the most common presentation of PCa in dialysis patients.^14^, ^15^ Since localized PCa is asymptomatic, it is likely that the majority of the cases, especially those in the low‐risk group, were diagnosed during screening. Despite the controversy around screening with PSA even in the general population, dialysis patients who are getting evaluated for a KT undergo screening with PSA at a very high rate of 89% during their evaluation.^16^ A multicenter study of 3782 candidates getting evaluated for KT, 1376 (36.4%) did not undergo PSA screening, compared to 2406 (63.6%) who underwent PSA screening, and had demonstrated that screening with PSA delayed and decreased chances of getting KT.^17^ Guidelines from the late 1990 s recommended curative treatment of PCa and 2–5 years of cancer‐free interval prior to KT.^18^ In a recent survey, 45% of transplant surgeons reported that definitive treatment of PCa was required before proceeding to KT, while AS was a viable option in 67% of responders.^16^ These screening and treatment practices are more aggressive than in the general population, and are based on the concern of untreated PCa progression due to immunosuppression but need to be carefully examined in light of recent evidence from population‐based studies that examined the relationship between immunosuppression and PCa. Our finding of a high proportion of dialysis patients with low‐risk PCa (>75%) undergoing a definitive treatment prior to receiving a KT likely reflects an aggressive approach to treatment, driven by the concern of untreated PCa progression after immunosuppression initiation.

A few recent studies using Surveillance, Epidemiology, and End Results (SEER)‐Medicare data found that solid organ transplant recipients, including those with KT, are not at increased risk either for developing PCa or for dying from PCa, compared to non‐transplanted men.^19^, ^20^, ^21^ Transplant recipients in these population‐based cohorts, such as our study, face two detection biases. They would have been screened at higher rates prior to and after transplant, which would have decreased and increased the risk of PCa posttransplant, respectively, and must have confounded the results. However, in dialysis patients, competing risk for mortality from other causes, which results in 3–4 times higher mortality than the general population, is very important in decision‐making for treatment. In the general population, a recent single‐center study evaluated the inclusion of competing risk for mortality among candidates with low‐risk PCa, who were under AS, to discuss conversion to WW when the disease was recategorized. They identified that a triad of age > 75, a Charlson comorbidity index (CCI) of >3, and ≤ Gleason Grade 2 (GG2) predicted a 14‐fold high non‐PCa mortality than PCa mortality and hence criteria for consideration for WW.^22^ Based on this study's recommendations since all patients in the dialysis cohort in our study with low‐risk PCa carried a CCI of >2 (due to dialysis status)^23^ and likely had a < GG2 PCa, they would have met criteria for WW. A simulation study of 10,000 hypothetical KT‐eligible candidates with low‐risk PCa concluded that quality‐adjusted life years and KT rates were highest for AS and immediate eligibility strategy for KT.^24^ A recent expert opinion recommendation from the AST/ASTS for low‐risk PCa recommends AS and immediate approval for KT.^25^ Since KT improves the overall mortality of dialysis patients, we support that consideration needs to be given to managing low‐risk PCa among dialysis patients by AS.

Societal guidelines for low‐risk PCa recommend AS as the preferred option for patients with a life expectancy of >10 years and WW for those with a life expectancy of either <5 or < 10 years.^3^, ^4^, ^5^ Accordingly, in the general population, a recent SEER‐based study of men with low‐risk PCa (*N* = 50,302) and intermediate risk (*N* = 81,836), Mahal et al., demonstrated that rates of definitive treatment had dropped from 2010–2015 (radical prostatectomy decreased from 47.4% to 31.3% and radiotherapy from 38.0% to 26.6%).^26^ Rates of definitive treatment for low‐risk PCa in non‐ESKD patients in our study showed a similar pattern, 72% in the 2004–2010 period and 48% in the 2011–2015 period. For dialysis patients with low‐risk PCa, on the contrary, these rates trended upwards, 54% in 2004–2010 and 63% in 2011–2015. These findings illustrate that there is a delay in shifting from definitive treatment to AS/WW among dialysis patients.

Our study has many strengths. We used the NCCN risk category framework to explore treatment patterns and mortality among dialysis patients with low and intermediate risk, which was not done previously to our knowledge. We used a population‐based study to identify a large cohort of dialysis patients, representative of the US. Our findings should be interpreted considering the following limitations. Circumstances that led to the diagnosis of PCa were not explored. We inferred that the lack of definitive treatment for PCa indicated AS or WW, which may not be true in a small number of cases, but this classification has previously been justified in the literature.^27^, ^28^ Despite the population‐based nature and use of multiple years' data, the number of deaths due to PCa was too small to conduct more detailed analyses but the number of deaths from any cause was high enough to conduct comparative analyses. In addition, given the small number of dialysis patients who received KT after a diagnosis of PCa, we were unable to compare time to KT or death between those who did and did not receive definitive treatment for PCa prior to KT. Since our cohort includes patients located in SEER regions in the United States, caution is advised in the extrapolation of these results to patients from other states and countries, where practices for screening for PCa and ESKD may be different. In addition, our study participants were all Medicare fee‐for‐service beneficiaries and hence may not be applicable to managed care beneficiaries.

In conclusion, despite high mortality among dialysis patients, they receive definitive treatments at a high rate and this pattern has not changed in recent years. Since very few patients in the low‐risk group die of PCa in dialysis or KT, AS or WW should be strongly considered during the shared decision‐making process, even in those who are being evaluated for KT. Concerted efforts from all the stakeholders and disciplines involved in managing this vulnerable population are needed to confirm our findings and incorporate AS/WW as a viable treatment option.

## AUTHOR CONTRIBUTIONS

**Nagaraju Sarabu:** Conceptualization (lead); funding acquisition (lead); investigation (lead); methodology (lead); project administration (lead); supervision (lead); writing – original draft (lead); writing – review and editing (lead). **Weichuan Dong:** Data curation (lead); methodology (lead); visualization (lead); writing – review and editing (supporting). **Al W. Ray:** Conceptualization (supporting); writing – review and editing (equal). **Austin Fernstrum:** Conceptualization (equal); writing – review and editing (equal). **Megan Prunty:** Writing – review and editing (equal). **Lee E. Ponsky:** Conceptualization (equal); writing – review and editing (equal). **Jonathan E. Shoag:** Conceptualization (equal); writing – review and editing (equal). **Vahakn B. Shahinian:** Conceptualization (equal); methodology (equal); writing – review and editing (equal). **Krista L. Lentine:** Writing – review and editing (equal). **Siran M. Koroukian:** Conceptualization (equal); methodology (equal); project administration (equal); resources (equal); supervision (equal); writing – review and editing (equal).

## FUNDING INFORMATION

Supported by intramural internal funding from University Hospital Research Department.

## CONFLICT OF INTEREST

Dr. Koroukian and Dr. Dong are supported by National Cancer Institute, Case Comprehensive Cancer Center (P30 CA043703), and the American Cancer Society (132678‐RSGI‐19‐213‐01‐CPHPS), and by contracts from Cleveland Clinic Foundation, including a subcontract from Celgene Corporation. In the past 36 months, Dr. Koroukian was also supported by grants from the Centers for Disease Control and Prevention (U48 DP005030‐05S1 and U48 DP006404‐03S7); the National Institutes of Health (R15 NR017792, and UH3‐DE025487); the American Cancer Society (RWIA‐20‐111‐02 RWIA). Dr. Lentine is a consultant for CareDx and is on the speakers' bureau for Sanofi.

## Supporting information


Appendix S1.

## Data Availability

The data underlying this article were provided by Surveillance, Epidemiology, and End Results by permission. Data will be shared on request to the corresponding author with the permission of Surveillance, Epidemiology, and End Results.
