# Supplementary material for: Treatment patterns and survival of low and intermediate‐risk prostate cancer in end‐stage kidney disease: A retrospective population cohort study
Source: Cancer Med. 2023 Jan 16;12(7):7941–50. doi: 10.1002/cam4.5571 (PMC10134264; doi:10.1002/cam4.5571)
Supplement: Supplementary file 1 — Appendix S1. [file CAM4-12-7941-s001.docx]

**Identification of dialysis and kidney transplant patients:**

**Transplant patients:** Those who had a billing claim with kidney transplant-related codes (see below) in the 365 days window, before prostate cancer diagnosis, and did not have any dialysis claim after the kidney transplant.

**Dialysis patient:** Those who had a billing claim with chronic renal failure-related codes (see below) and a billing claim with dialysis-related codes (see below) in the 365 days window, before prostate cancer diagnosis.

**ICD diagnosis codes**

CRF: any subcategories starting with ICD 9 codes: 582, 585, 586, 587, 403, 404, and 250.4 or ICD-10 codes N18.3, N 18.4, N18.5 and N18.6

Kidney Transplant: ICD 9: V420; ICD 10: Z940

**CPT/HCPCS codes**

**Dialysis:** '90918', '90919', '90920', '90921', '90922', '90923', '90924', '90925', '90935', '90936', '90937', '90945', '90946', '90947', '90951', '90952', '90953', '90954', '90955', '90956', '90957', '90958', '90966', '90967', '90968', '90969', '90970', '90971', '90972', '90973', '90974', '90975', '90976', '90977', '90978', '90979', '90980', '90981', '90982', '90983', '90984', '90985', '90988', '90989', '90990', '90991', '90994', '99559'

**Kidney Transplant:** "50360", "50365"

**ICD procedure codes**

**Dialysis:** "3995", "5498"
**Kidney Transplant:**"5569"

**Revenue center codes**

**Dialysis:** any codes starting with 082, 083, 084, 085, 086, 087
